# The clinical value of vHIT saccadic parameters in the diagnosis and management of central vestibular disorders: a narrative review

**DOI:** 10.3389/fneur.2026.1827448

**Published:** 2026-07-24

**Authors:** Li Qian, Yang Shunjia, Wang Hulin, Chen Xu, Tang Enjie, Li Ling

**Affiliations:** 1 Department of Neurology, 925 Hospital of the PLA Joint Logistics Support Force, Guiyang, Guizhou, China; 2 925 Hospital of the PLA Joint Logistics Support Force, Guiyang, Guizhou, China; 3 General Surgery Department, 925 Hospital of the PLA Joint Logistics Support Force, Guiyang, Guizhou, China

**Keywords:** acute vestibular syndrome, central vestibular disorders, clinical diagnosis, saccades, video head impulse test

## Abstract

Central vestibular disorders significantly impair human balance and spatial orientation, and are associated with high rates of disability and mortality. Precise diagnosis and management are crucial for improving patient prognosis. As an advanced vestibular function assessment technique, the video head impulse test (vHIT) has been widely applied in clinical practice due to its non-invasive, accessible, and quantitative properties. Although the vestibulo-ocular reflex (VOR) gain serves as the core metric of vHIT, it presents notable limitations due to the influence of various factors such as testing techniques and patient compliance. Relying solely on the VOR gain poses a challenge to reliably differentiate central from peripheral vestibular disorders in acute vestibular syndrome. This limitation may lead to misdiagnosis when distinguishing conditions such as vestibular neuritis from cerebellar ischemic stroke. In recent years, clinical research has increasingly focused on the quantitative characteristics of compensatory saccades. When interpreted together with VOR gain and clinical context, multidimensional saccadic parameters, including latency, amplitude, symmetry, and dispersion, may provide complementary information on vestibular compensation and central ocular motor control. This narrative review provides a clinically oriented synthesis of the potential value of multidimensional saccadic parameters in differential diagnosis, selected lesion-localization contexts, and compensation monitoring, while emphasizing that these parameters should be interpreted together with VOR gain and broader clinical findings.

## 1 Introduction

Vertigo is a common and complex clinical symptom commonly related to dysfunction of the vestibular system, severely impacting patients’ daily lives and social functioning. Central vestibular disorders are an important cause of vertigo and balance impairment (1, 2). With the rapid development of vestibular function testing technologies, the video head impulse test (vHIT) has become increasingly important in the clinical management of vestibular diseases because of its non-invasive, accessible, quantitative, and rapid features (3–5). Some peripheral vestibular disorders, particularly vestibular neuritis, commonly present with reduced vestibulo-ocular reflex (VOR) gain accompanied by corrective saccades, whereas selected central vestibular disorders may show normal or mildly abnormal VOR gain with atypical saccadic patterns (6, 7).

Although the VOR gain is widely used as the primary quantitative metric of vHIT, its clinical interpretation is highly susceptible to multiple confounding factors (8). Factors such as inconsistent testing techniques by examiners, goggle strap tightness, and algorithmic differences among devices may lead to significant variations in the calculated VOR gain values (9), while age-related neurodegenerative changes may also cause a non-specific decline in the VOR gain. These limitations result in a high risk of misdiagnosis in acute vestibular syndrome (10). These limitations highlight the need to interpret VOR gain together with other vHIT-derived features and clinical findings (11). Corrective saccades are not disease-specific and may occur in both peripheral and central vestibular disorders. However, when interpreted as multidimensional patterns, including latency, amplitude, symmetry, dispersion, direction, and their relationship with VOR gain, they may provide complementary information on vestibular compensation and central ocular motor control (12). Furthermore, the Perez and Rey Score (PR Score) has been proposed as a quantitative measure of the temporal organization or dispersion of corrective saccades during vHIT (13). It has been explored as a potential indicator of vestibular compensation, particularly in peripheral vestibular disorders, although its interpretation in central vestibular disorders remains insufficiently validated (13, 14). Based on these considerations, this narrative review focuses on three clinically relevant questions. Specifically, we discuss how saccadic parameters may complement VOR gain in differentiating central from peripheral vestibular disorders, how specific saccadic patterns may assist lesion localization in selected central vestibular disorders, and how dynamic changes in saccadic parameters may inform rehabilitation monitoring and vestibular compensation.

## 2 Review scope and literature selection

This article was designed as a clinically oriented narrative review addressing the interpretation of vHIT saccadic parameters in central vestibular disorders. To support the narrative synthesis, relevant literature was primarily identified through targeted searches of PubMed, Embase, and Web of Science from database inception to March 2026. Search concepts were organized around four domains: video head impulse testing and VOR gain; corrective, compensatory, or corrective saccades; central vestibular disorders and acute vestibular syndrome; and clinically relevant comparator or overlapping conditions, including posterior circulation stroke, AICA/PICA infarction, vestibular neuritis, labyrinthitis, vestibular migraine, cerebellar disorders, and vestibular rehabilitation. Priority was given to original clinical studies, consensus statements, and major reviews that directly informed the interpretation of vHIT gain, saccadic latency, amplitude, symmetry, dispersion, lesion localization, or rehabilitation monitoring. Studies with limited relevance to vHIT interpretation, exclusively experimental or animal data, or peripheral vestibular disorders outside the scope of this review were not emphasized. Because the aim was interpretive clinical synthesis rather than exhaustive evidence pooling, no formal risk-of-bias assessment or meta-analysis was performed.

## 3 Evolution of vHIT metrics

The vHIT evaluates VOR function, and provides objective evidence for the diagnosis and management of vestibular disorders. In clinical practice, the VOR gain is currently widely used to assess the integrity and functional impairment of the semicircular canals and their associated vestibular pathways (15). However, accumulating evidence highlights the synergistic value of combining the VOR gain with compensatory saccades. Joint analysis of both metrics may comprehensively reflect the overall functional status and extent of damage of the vestibular system, thereby providing critical support for clinical diagnosis, disease assessment, and rehabilitation guidance (16).

### 3.1 Limitations of VOR gain

Although the VOR gain remains a fundamental quantitative metric in vHIT, its isolated application has inherent limitations, particularly when evaluating central vestibular disorders. Methodologically, the calculation of the VOR gain is highly susceptible to test artifacts, including inconsistent examiner techniques, goggle slippage, and algorithmic variations among different eye-tracking devices (9, 17). Clinically, relying exclusively on VOR gain may increase the risk of diagnostic ambiguity or misclassification in patients presenting with acute vestibular syndrome. Abnormal VOR gain reductions can manifest in both peripheral vestibular disorders and certain central infarctions (such as posterior circulation stroke), and thus using this single metric is insufficient for reliable topographical differentiation (18).

### 3.2 Advantages of corrective saccades

Corrective saccades may provide complementary information beyond VOR gain alone, particularly regarding the timing, direction, amplitude, and organization of refixation responses. However, their detection and quantification remain method-dependent and should be interpreted in light of recording quality, device characteristics, analytic algorithms, head impulse technique, and patient cooperation (9, 11, 19). Based on their temporal occurrence, saccades are classified into overt (occurring after the head impulse) and covert (occurring during the head impulse) types (19). Monitoring the dynamic changes in their frequency and velocity provides insights into the progression of vestibular recovery (20, 21).

### 3.3 Quantifiable parameters of saccades

While compensatory saccades are seen in both peripheral and central vestibular dysfunction, their core parameters (latency, amplitude, symmetry, dispersion) may show clinically informative patterns that can support the assessment of selected central vestibular disorders. Latency describes the interval between the head impulse and the onset of a corrective saccade. Prolonged or highly variable latency may indicate altered timing of the corrective response, but it is not specific to a single anatomical pathway and should be interpreted together with saccade amplitude, direction, dispersion, VOR gain, and clinical findings (7). Saccadic dysmetria, characterized by overshooting or undershooting of the target, may reflect impaired calibration of ocular motor responses rather than a disease-specific sign (22, 23). Furthermore, side-to-side comparison of corrective saccadic responses may help characterize asymmetry between the affected and unaffected sides; however, such asymmetry should be interpreted in conjunction with VOR gain, lesion context, and clinical findings rather than as an independent lateralizing sign (18, 24). Finally, the PR Score has been proposed as a quantitative measure of the temporal organization or dispersion of corrective saccades during vHIT (13, 25). Higher PR scores indicate greater temporal dispersion of corrective saccades, whereas lower scores indicate more temporally clustered responses. In peripheral vestibular hypofunction, especially vestibular neuritis, changes from scattered overt saccades toward more clustered covert or organized saccades have been explored as a marker of vestibular compensation, sometimes even when VOR gain shows limited recovery (13, 14, 26). However, the interpretation of PR score in central vestibular disorders remains insufficiently validated.

## 4 Diagnostic value in central disorders

Central vestibular disorders encompass a variety of conditions, including brainstem infarction, hemorrhages, inflammation, demyelination, and brain tumors (27). The core pathological mechanism involves lesions affecting the brainstem, cerebellum, and their connecting pathways, which leads to central vestibular integration dysfunction (28). Because the clinical presentations of these diseases overlap significantly with those of peripheral vestibular disorders, accurate differentiation using only symptomatology and structural neuroimaging is frequently inadequate (29). As complementary quantitative features of vHIT, corrective saccade parameters may provide additional diagnostic and localizing information in selected central vestibular disorders when interpreted together with VOR gain, bedside ocular motor findings, and clinical context (7, 30). However, these findings must always be interpreted in conjunction with central vertigo risk factors and appropriate neuroimaging studies.

### 4.1 Differentiating central from peripheral vestibular disorders

The vHIT profiles of central and peripheral vestibular disorders may show clinically informative differences, although substantial overlap exists. In comparisons between posterior circulation stroke and vestibular neuritis, vestibular neuritis has been reported to show marked ipsilesional VOR gain reduction, with ipsilesional corrective saccades that tend to be earlier, larger, more prevalent, faster, and more asymmetric than those observed in posterior circulation stroke (6, 18). These findings support the value of combining VOR gain with corrective-saccade metrics, but they should be interpreted as study-specific comparative patterns rather than universal diagnostic criteria.

Clinical evidence suggests that relying exclusively on the VOR gain may lead to diagnostic ambiguity or misclassification in selected acute vestibular syndrome (AVS) contexts. In AVS, a reduced VOR gain can occur in both vestibular neuritis and selected posterior circulation strokes, including AICA or PICA territory infarctions, particularly when peripheral vestibular structures or central VOR pathways are involved (4, 6, 12, 31). Therefore, VOR gain alone is insufficient for reliable central–peripheral differentiation in all AVS cases. Integrating corrective saccade parameters may help reduce these diagnostic ambiguities when interpreted together with bedside ocular motor findings and clinical context. For instance, patients with PICA infarction have been reported to show smaller, more bilateral, and less asymmetric corrective saccades than those observed in vestibular neuritis (10, 24).

The diagnostic challenge in AVS often hinges on the differentiation between AICA territory infarctions and labyrinthitis, both of which can present with concomitant hearing loss and vestibular dysfunction. A recent comparative study reported that a bilaterally positive vHIT was more frequent in AICA infarction than in labyrinthitis (31). When combined with hearing symptoms, vascular risk assessment, and other bedside findings, this pattern may provide a supportive clue for AICA territory infarction, but it should not be interpreted as a standalone diagnostic marker.

Furthermore, prolonged or highly variable saccadic latency and atypical saccadic dispersion may raise suspicion of central vestibular involvement, but these findings are not specific to a single anatomical pathway or disease entity (7, 12, 30). In isolated nodular/heminodular infarction, preserved horizontal and vertical aVOR integrity has been reported and may provide a supportive clue in the appropriate clinical context, although this finding should not be interpreted as a standalone criterion for central–peripheral differentiation (32). In addition, a broader set of saccade metrics combined with VOR gain may help detect vestibular stroke in selected clinical settings (30).

Beyond vascular events, saccadic metrics may also provide supportive information in selected neurodegenerative and metabolic disorders. Patients with multiple system atrophy have been reported to exhibit more frequent reversed and perverted corrective saccades, as well as greater horizontal–vertical gain discrepancies, compared with patients with Parkinson’s disease, which may aid differential diagnosis (33). Metabolic conditions, such as Wernicke’s encephalopathy, can also manifest early vHIT abnormalities, including bilateral horizontal VOR deficits and pathological gaze-evoked nystagmus before overt encephalopathic signs become apparent (34).

**Figure 1:**
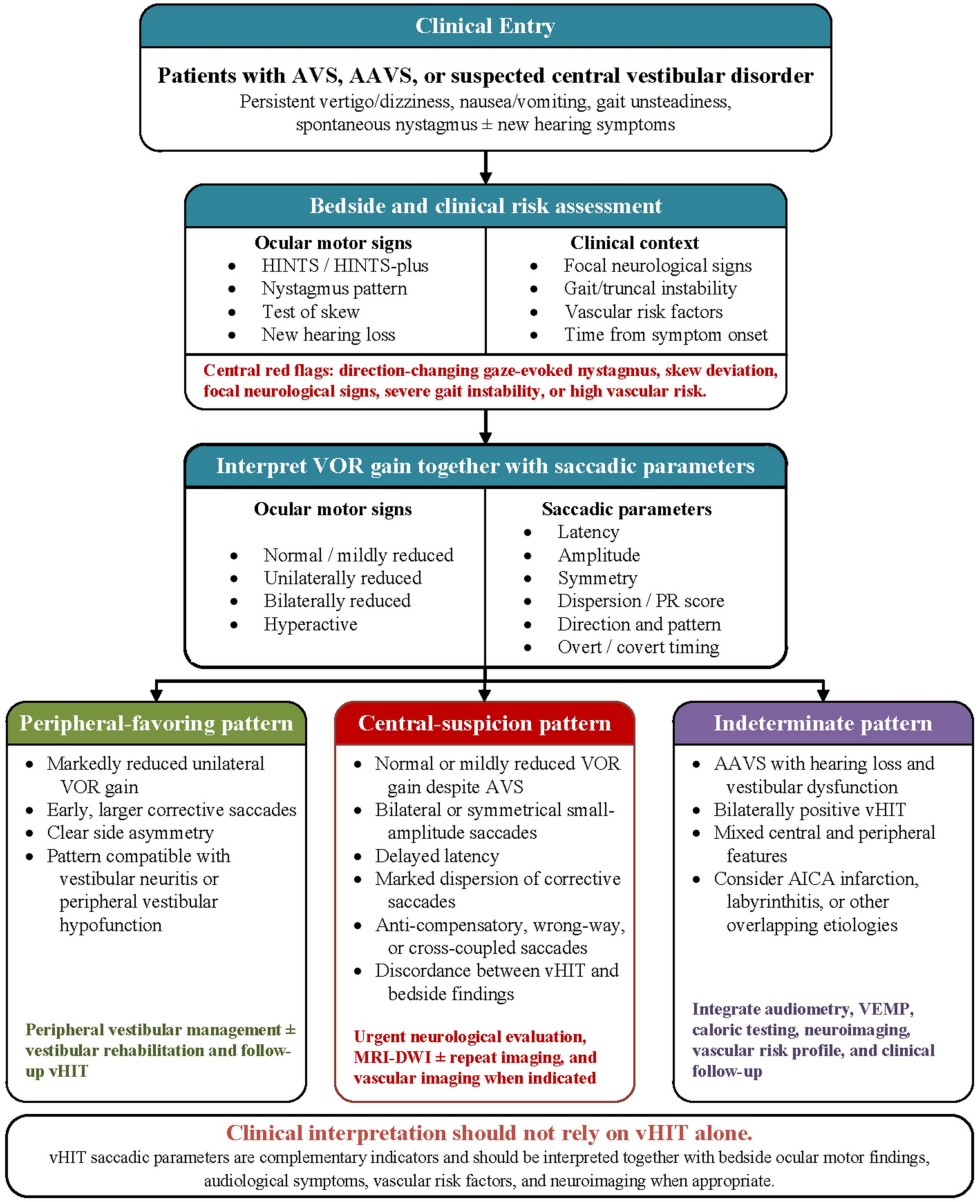
Proposed clinical interpretation pathway. The pathway summarizes how vHIT findings may be interpreted within the broader clinical evaluation of AVS, AAVS, or suspected central vestibular disorders. Initial assessment should include bedside ocular motor examination, HINTS/HINTS-plus, nystagmus pattern, skew deviation, new hearing loss, focal neurological signs, and vascular risk factors. vHIT interpretation should integrate both VOR gain and multidimensional saccadic features, including latency, amplitude, symmetry, dispersion, and atypical saccadic patterns. This pathway is intended as a clinical interpretation framework rather than a validated diagnostic algorithm.

To translate these pattern-based findings into clinical practice, Figure 1 summarizes a proposed interpretation pathway for integrating vHIT saccadic parameters with bedside ocular motor examination, audiological findings, vascular risk factors, and confirmatory testing in patients with AVS, AAVS, or suspected central vestibular disorders.

### 4.2 Topographical localization of central pathologies

Specific focal pathologies within the central vestibular system may be associated with relatively characteristic, although overlapping, saccadic abnormalities, which may provide complementary localizing information when early neuroimaging is unrevealing or clinically inconclusive. Brainstem involvement may disrupt saccade initiation and ocular motor processing pathways, clinically presenting as highly variable latencies and impaired saccade initiation in selected cases (35). Conversely, cerebellar dysfunction may compromise the accuracy and temporal organization of eye movements, manifesting as saccadic dysmetria or increased dispersion, which presents as either overshooting or undershooting the target (7, 23).

Selected central pathologies have been associated with relatively characteristic vHIT patterns. Diffuse cerebellar disorders have been reported to present with hyperactive VOR gain accompanied by anti-compensatory (back-up) saccades, occasionally producing cross-coupled vertical saccades during horizontal impulses, which may indicate diffuse cerebellar involvement. Infarction in the PICA or superior cerebellar artery (SCA) territories, as well as floccular disorders, may demonstrate bilaterally positive corrective saccades alongside mildly reduced VOR gain in selected reports. Unilateral vestibular nucleus infarctions have been reported to show large-amplitude saccades ipsilaterally and covert saccades contralaterally, with bilateral VOR gain reduction on quantitative HIT. Additionally, selected cerebellar disorders may show hyperactive VOR gain combined with anti-compensatory saccades (7). These reported patterns may provide additional clues for anatomical localization in selected clinical contexts (see Table 1).

**Table 1:** Representative vHIT patterns reported in selected central and peripheral vestibular disorders.

Anatomical origin	Pathological subtype	Reported VOR gain characteristics	Reported saccadic features in selected studies	References
Central vestibular disorders	PICA/SCA territory cerebellar stroke	Clinical horizontal HIT is often normal; quantitative HIT may reveal mild bilateral reduction in horizontal VOR gain in selected reports.	Small corrective saccades with minimal left–right gain asymmetry may accompany the mild gain reduction.	Choi et al. (7) and Chen et al. (10)
	AICA territory stroke/combined peripheral–central vestibular involvement	Clinical HIT may be positive or normal; quantitative HIT may show ipsilesional and/or contralesional horizontal VOR gain reduction.	Corrective saccades may occur when inner-ear and central VOR pathways are involved; interpretation should integrate hearing symptoms and vascular risk factors.	Choi et al. (7), Celebisoy (12), and Kim et al. (31)
	PICA infarction	Horizontal/anterior canal VOR gain may be more symmetric and relatively preserved than in vestibular neuritis, although gain can vary with lesion extent.	Corrective saccades are reported as smaller, more bilateral, and less asymmetric than those in vestibular neuritis.	Nam et al. (24)
	Vestibular nucleus lesion/infarction	Unilateral vestibular nucleus lesions can produce ipsilesional clinical HIT abnormality and bilateral horizontal VOR impairment on quantitative HIT.	Covert saccades during head impulses toward the intact side have been described in selected cases.	Choi et al. (7)
	Flocculus or nucleus prepositus hypoglossi lesions	Restricted flocculus or nucleus prepositus hypoglossi lesions can produce positive clinical and quantitative horizontal HITs.	Direction-specific positive HIT patterns may differ from typical peripheral vestibular loss.	Choi et al. (7)
	Diffuse cerebellar dysfunction	Horizontal HIT may show hyperactive VOR or other abnormal gain patterns in diffuse cerebellar disorders.	Reversed/anti-compensatory back-up saccades and cross-coupled vertical corrective saccades during horizontal HIT have been reported.	Choi et al. (7)
	Isolated nodular/heminodular infarction	Quantitative vHIT has shown preserved horizontal and vertical aVOR integrity in isolated nodular or heminodular strokes.	No disease-specific corrective-saccade criterion has been established for this subgroup.	Lee et al. (32)
	Multiple sclerosis	VOR gain may be abnormal in selected patients with MS, particularly when brainstem pathways are involved.	Oculomotor and saccadic abnormalities have been reported; vHIT may help detect brainstem dysfunction in selected MS patients.	Evangelista et al. (42) and Pavlović et al. (43)
	Vestibular migraine, interictal	VOR gains are often largely normal in reported interictal cohorts.	Catch-up saccades may be observed, but no diagnostic saccade threshold is established.	Adachi et al. (44)
Peripheral vestibular disorders	Vestibular neuritis	Marked ipsilesional VOR gain reduction is typical.	Ipsilesional catch-up saccades are typically earlier, larger, more prevalent, and faster than those observed in posterior circulation stroke.	Nham et al. (18)
	Bilateral vestibulopathy	Bilateral horizontal angular VOR gain <0.60 on vHIT is part of the Bárány Society diagnostic criteria.	Corrective saccades may be present, but the consensus diagnostic criteria are based on VOR reduction rather than a saccade threshold.	Strupp et al. (45)

This table summarizes representative vHIT patterns reported in selected central and peripheral vestibular disorders. These findings should be interpreted as supportive clinical clues rather than validated diagnostic criteria. Except for established diagnostic criteria, such as the Bárány Society criterion for bilateral vestibulopathy, numerical thresholds are intentionally avoided because reported values vary across cohorts, devices, testing protocols, and analytic definitions.

### 4.3 Monitoring rehabilitation and prognosis

Rehabilitation of vestibular disorders generally aims to promote vestibular compensation and functional recovery. In central vestibular disorders, however, the mechanisms of compensation are more heterogeneous, and direct longitudinal evidence validating corrective-saccade parameters as prognostic markers remains limited. Because traditional efficacy evaluations rely heavily on subjective symptom descriptions, dynamic changes in corrective saccade parameters may provide complementary information for monitoring vestibular compensation and functional recovery, although their prognostic thresholds and longitudinal validity remain insufficiently established (36, 37). Notably, corrective saccades may be present even when VOR gain is normal or before clear gain reduction is detected, indicating that saccadic parameters can provide information beyond VOR gain alone (38).

Most longitudinal evidence in this area comes from vestibular neuritis, acute unilateral vestibulopathy, or unilateral peripheral vestibular hypofunction. In these conditions, patients who recover or compensate may show changes from scattered overt saccades toward more covert, clustered, or temporally organized saccadic responses, sometimes even when VOR gain shows limited recovery (20, 36, 37, 39). In cases of vestibular neuritis, prominent corrective saccades may change over time together with VOR gain recovery and clinical improvement (20, 21, 39). Persistent abnormal saccadic patterns after the acute phase may reflect ongoing or incomplete vestibular compensation in peripheral vestibular hypofunction, but their prognostic significance in central vestibular disorders remains insufficiently established (36, 37). Furthermore, changes in corrective saccades may parallel functional vestibular recovery even when numerical VOR gain remains incompletely normalized, suggesting that saccadic dynamics may serve as a complementary, indirect marker of compensation rather than a validated prognostic endpoint (36, 37, 40).

Evidence from vestibular migraine should be interpreted separately from rehabilitation studies. Vestibular function tests, including vHIT and caloric testing, have been explored for their clinical implications in vestibular migraine, but available evidence does not establish post-treatment VOR gain normalization as a general marker of clinical resolution (41).

## 5 Conclusion and future perspectives

Driven by the rapid technological evolution in vestibular function testing, the vHIT has become an important auxiliary tool in the clinical management of central vestibular disorders (30). Compared to the traditional sole reliance on the VOR gain, the quantifiable parameters of compensatory saccades may provide complementary diagnostic information. These metrics can partly address the limitations of relying on VOR gain alone, thereby improving the interpretation of central versus peripheral etiologies in selected clinical settings (18). Moreover, these parameters may support lesion localization and rehabilitation monitoring in selected clinical contexts, although further validation is required before they can be adopted as standardized diagnostic or prognostic criteria.

Several limitations should be considered when interpreting vHIT saccadic parameters. Corrective saccades are not disease-specific and may occur in both peripheral and central vestibular disorders; therefore, their diagnostic value depends on pattern-based interpretation rather than their mere presence. In addition, saccadic parameters may be influenced by device type, recording quality, head impulse technique, analytic algorithms, patient cooperation, and the timing of assessment after symptom onset. Current evidence is also limited by heterogeneous study populations, variable definitions of saccadic metrics, and the lack of universally validated diagnostic thresholds. Accordingly, vHIT saccadic parameters should be regarded as complementary clinical indicators and interpreted together with VOR gain, bedside ocular motor findings, audiological symptoms, vascular risk factors, and neuroimaging when appropriate.

Despite these potential advantages, most evidence is derived from studies of peripheral vestibular disorders, and extrapolation to central vestibular diseases calls for careful clinical interpretation and contextual judgment. Given the overlap in clinical manifestations and vHIT findings between central and peripheral vestibular disorders, vHIT results must be interpreted in conjunction with clinical examination, nystagmography, caloric testing, audiological assessment, and neuroimaging. Standardized testing protocols and definitive parameter thresholds have yet to be widely accepted, and the precise clinical significance of complex metrics, such as the dispersion coefficient, requires rigorous validation. Future efforts should prioritize large-scale, multicenter clinical trials designed to optimize these detection standards and evaluate whether robust and clinically useful saccadic patterns can be identified for different central pathologies. Furthermore, the integration of advanced computational methodologies and multi-dimensional statistical modeling is essential for processing the complex datasets generated from these saccadic metrics. Specifically, the application of robust statistical programming and data visualization techniques to generate polar plots and multidimensional dispersion models is critical for accurately interpreting PR scores and other variance metrics. Merging these analytical frameworks with emerging artificial intelligence technologies will facilitate the development of sophisticated diagnostic and prognostic models, thus advancing the paradigm of precision medicine in neurotology.
